# Phase I First-in-Human Dose Escalation Study of the oral Casein Kinase 1α and Cyclin Dependent Kinase 7/9 inhibitor BTX-A51 in advanced MDS and AML

**DOI:** 10.21203/rs.3.rs-4954060/v1

**Published:** 2024-10-15

**Authors:** Brian Ball, Wenbin Xiao, Gautam Borthakur, Le Xuan Truong Nguyen, Melissa Valerio, Avanthika Venkatachalam, Guido Marcucci, Anthony Stein, Dung Luong Thai, David Cook, Kyle Chan, Sonali Persaud, Ross Levine, Omar Abdel-Wahab, Yinon Ben-Neriah, Eytan Stein

**Affiliations:** City of Hope National Medical Center; Memorial Sloan Kettering Cancer Center; The University of Texas MD Anderson Cancer Center; City of Hope National Medical Center; City of Hope Medical Center; The Lautenberg Center for Immunology and Cancer Research; City of Hope Medical Center; City of Hope National Medical Center; Edgewood Oncology; Edgewood Oncology; BioTheryx; Memorial Sloan Kettering Cancer Center; Memorial Sloan Kettering Cancer Center; Memorial Sloan Kettering Cancer Center; The Lautenberg Center for Immunology and Cancer Research, Institute of Medical Research Israel-Canada; Memorial Sloan Kettering Cancer Center

## Abstract

BTX-A51, a first-in-class oral small molecule inhibitor of casein kinase 1α (CK1α) and cyclin dependent kinase (CDK) 7 and 9, induces apoptosis of leukemic cells by activating p53 and inhibiting expression of *Mcl1*. Here, we report on the results of the phase 1 clinical trial of BTX-A51 in patients with relapsed or refractory AML and MDS. Thirty-one patients were enrolled into 8 dose-escalation cohorts at BTX-A51 doses ranging from 1mg to 42mg dosed three days/week for 21 or 28 days out a 28-day cycle. The recommended phase 2 dose was 21mg dosed three days/week for 4 weeks of a 28-day cycle. BTX-A51 increased expression of p53 and reduced expression of MCL1 and RNA polymerase II phosphorylation on pre- and post-treatment immunocytochemistry studies. Overall, 3 patients (10%) experienced complete remission with incomplete count recovery (CRi). All 3 responding patients had *RUNX1* mutations and the CR/CRi rate for *RUNX1*-mutated patients receiving BTX-A51 at efficacious doses (11mg or higher) was 30%. Ex-vivo studies confirmed higher efficacy of BTX-A51 on *RUNX1*-mutated myeloblasts and demonstrate synergy with azacitidine and venetoclax. Although the overall efficacy was modest, this study lays the groundwork for future studies with improved patient selection and combination approaches.

## Introduction

Acute myeloid leukemia (AML) and high-risk myelodysplastic syndromes are genetically heterogeneous diseases associated with poor long-term survival.(1, 2) Despite most patients achieving a morphologic remission with frontline AML therapies, approximately 50% of patients still relapse.(3) For those without targetable *IDH* or *FLT3* mutations, effective treatment options for R/R AML are limited, and long-term survival rates are dismal. The 2-year overall survival (OS) for adult patients with R/R AML after intensive chemotherapy is < 30%.(4, 5) Among patients with high-risk MDS, failure of frontline hypomethylating agents is associated with especially poor outcomes with a median OS of 5.6 months.(6) Although much progress has been made towards understanding disease biology, relapsed or refractory (R/R) AML and MDS remain challenging diseases with few effective therapies.

Therapeutic targeting of the anti-apoptotic protein B-cell lymphoma-2 (BCL-2) with venetoclax in combination with hypomethylating agents or low-dose cytarabine induces high rates of complete remission in newly diagnosed elderly patients and those with R/R AML.(7–10) However, failure of venetoclax is associated with especially poor outcomes with a median OS of 2.4 months.(11) Resistance to venetoclax commonly arises as a result of either overexpression of other antiapoptotic BCL2 family members such as *MCL1* and *BCL-XL* or inactivation of P53. The antiapoptotic protein *MCL1* is overexpressed in AML cell lines resistant to venetoclax.(12) Similarly, the sensitivity of AML patients samples to venetoclax inversely correlates with the presence of a *TP53* mutation or low expression of *P53*.(13) In AML, P53 inactivation commonly results from overexpression of its negative regulators, *MDMX* and *MDM2*.(14–16)

BTX-A51 is an oral multi-kinase inhibitor having a unique set of target enzymes; it is a direct inhibitor of Casein Kinase 1α (CK1α) and cyclin dependent kinase 7 (CDK7) and CDK9. CK1α, a serine-threonine kinase, functions as a negative regulator of p53, partly by enhancing its binding by MDMX and MDM2 (15–17). CDK7 and CDK9 phosphorylate RNA polymerase II (Pol II) to enable transcriptional initiation and elongation, particularly at large clusters of transcriptional enhancers termed super-enhancers (SE) (18, 19). Treatment of AML cells with BTX-A51 robustly stabilizes and activates the p53 protein via a combined action of CK1α inhibition and shutdown of MDM2 expression, and at the same time it preferentially suppresses SE-driven transcription of key oncogenes such as *MYC, MCL1*, and *MYB*, enabling selective apoptosis of leukemic stem cells (20). The combination of CK1α and CDK7 and CDK9 inhibition was synergistic and prolonged survival in multiple genetic and patient-derived xenograft AML models (20). BTX-A51 also demonstrated a “rapid-hit” mechanism with near-complete elimination of CDK7/CDK9 mediated RNA Pol II phosphorylation and *Mdm2* expression after only 5 minutes of treatment in a mouse AML cells (20). This first-in-human phase I dose-escalation study aimed to determine the safety and recommended phase 2 dose. Secondary endpoints included pharmacokinetic (PK) and pharmacodynamic (PD) properties, and preliminary antitumor activity of BTX-A51 in patients with R/R AML and high-risk MDS.

## Patients and Methods

### Patients

Eligible patients were age ≥ 18 years or older, with a histologically confirmed diagnosis of AML or MDS according to the World Health Organization criteria,(21) and had R/R disease. Those with MDS were high or very high risk by the Revised International Prognostic Scoring System (IPSS-R) and had either not responded to or progressed on hypomethylating agent therapy.(22) Patients had an Eastern Cooperative Oncology Group (ECOG) performance status of 0 to 2, a white blood cell (WBC) count of 25 × 10^9^ /L or less, and adequate organ function, defined as a serum creatinine ≤ 1.5 × the institution’s upper limit of normal (ULN), total bilirubin ≤ 1.5 × ULN, AST/ ALT ≤ 2 × ULN. Additional eligibility criteria are described in the Supplementary Material.

### Study Design

This was a first-in-human, phase I, open-label, dose escalation study conducted at 3 centers: Memorial Sloan Kettering Cancer Center, The University of Texas MD Anderson Cancer Center, and City of Hope National Medical Center. The study utilized a hybrid accelerated titration with single patient cohorts and a Bayesian Optimal Interval (BOIN) to determine the maximal tolerated dose of BTX-A51 monotherapy. In the BOIN design, the maximum tolerated dose (MTD) was the dose for which the isotonic estimate of the toxicity rate was closest to the target toxicity rate of 0.3. The trial included 9 pre-planned dosing cohorts of BTX-A51 monotherapy at different dose levels and dosing schedules: 5 days per week for 21 out of 28 days (cohort 1) and 3 days per week for 21 out of 28 days (cohorts 2–7). A cohort of BTX-A51 dosed at 21mg for 3 days per week for 28 out of 28 days was subsequently explored. As CDK9 inhibitors and MDM2 inhibitors have led to tumor lysis syndrome (TLS) in advanced hematologic malignancies (23–26) and BTX-A51 demonstrated a “rapid-hit” mechanism,(20) the trial incorporated TLS prophylaxis during the first cycle. The TLS prophylaxis consisted of allopurinol starting 72 hours prior to dosing, intravenous fluids 24 hours prior to dosing, and TLS monitoring in the hospital or in the outpatient setting.

The study protocol was approved by the Institutional Review Boards at each participating center. All patients provided written informed consent before screening and enrollment. The study was conducted in accordance with the principles of the Declaration of Helsinki and Good clinical Practice guidelines. The study was registered at Clinicaltrials.gov (Identifier NCT04872166) prior to enrollment of the first patient. Study drug was provided by BioTheryx Inc, San Diego, California, USA, which also provided scientific, logistical and financial support for the trial.

### Assessments

The primary objective was to determine the safety, MTD, and recommended phase II dose of BTX-A51 based on the incidence of dose-limiting toxicities (DLTs) during cycle 1. Adverse events (AEs) were graded according to the Common Terminology Criteria for Adverse Events (CTCAE), version 5.0. DLTs were defined as nonhematologic toxic effects of grade 3 or higher according to the CTCAE version 5 or prolonged myelosuppression with persistence of ≥ grade 3 neutropenia or thrombocytopenia in the absence of leukemia at least 42 days after treatment initiation.

Secondary objectives included determining an estimate of preliminary efficacy, overall and event free survival and to evaluate the pharmacokinetics and pharmacodynamics of BTX-A51. Clinical efficacy was assessed by investigators according to European LeukemiaNet (ELN) guidelines in AML(27) and International Working Group (IWG) guidelines in MDS.(28) Pharmacokinetic profiling of BTX-A51 was assessed with serial peripheral blood collected pre-dose on days 1, 5, and 15 on days 1, 5, 6, 7, 8, and 15 during cycle 1. PK analysis used liquid-liquid extraction, followed by high-performance liquid chromatography/ mass spectrometry/ mass spectrometry analysis. Pharmacodynamic (PD) serum and peripheral blood (PB) samples were collected at baseline, on day 5 (after the first week of treatment) and weekly thereafter for cycle 1. p53 activation was measured by serum macrophage inhibitory cytokine-1 levels (MIC-1) levels before and after treatment with BTX-A51. Serum MIC-1 concentrations were measured using a commercially available ELISA kit (human growth differentiation factor 15 Quantikine, R&D Systems, Minneapolis, Minnesota), per the manufacturer’s instructions. Additionally, bone marrow specimens were collected during screening and at the end of cycle 1 for fluorescence immunohistochemistry by confocal laser-scanning microscopy to assess expression of Mcl1, RNA polymerase II, and p53. Primary CD34 + AML cells were washed in ice-cold PBS, fixed in 4% paraformaldehyde for 15 minutes and permeabilized in 0.5% Triton X-100 for 15 minutes. Non-specific epitopes were blocked with 5% bovine serum albumin (BSA) for 30 minutes. The cells were then stained with anti-MCL1, anti-p-Pol II, anti-p53, or anti-pH2AX antibodies. Secondary anti-mouse-Alexa 488 and anti-rabbit-Alexa 594 goat antibodies were purchased from Thermo Scientific. Cell images were acquired using a Zeiss confocal laser-scanning-microscope (Zeiss LSM 800). Nuclei were counterstained with ProLong Gold Antiface with DAPI (Molecular Probes, Invitrogen). Mutational profiling was performed on baseline bone marrow biopsy samples by next generation sequencing per institutional standards at each site.

### Ex vivo co-culture studies

Viably cryo-banked bone marrow cells obtained from patients with AML were retrieved, thawed and stained with anti-human CD45-FITC (clone 2D1, BioLegend, San Diego, California), anti-human CD34-APC (clone 581, BioLegend, San Diego, California) and anti-human CD117 BV785 (clone 104D2, BioLegend, San Diego, California). Viable CD45 + CD34 + CD117 + leukemic blasts were sorted by flow cytometry activated cell sorting and co-cultured with human umbilical vein endothelial cells transduced with an adenovirus gene, early region 4 encoded open reading frame-1 (E4ORF1) (HUVEC_AD52 cells, kindly provided by Shahin Rafii Laboratory)(29) in the presence of serum free medium StemSpan and cytokine cocktails (human SCF/FLT3L/TPO each 50 ng/ml and stemreginin R1 1mM) for 7 days. The cells were accutased, harvested, counted and stained for flow cytometry. CD34 positive blasts were enumerated. The inhibitors were added on day 1 and day 4.

### AML cell line studies

All cell lines were obtained from ATCC or DSMZ collection (MV-4–11: CRL-9591, THP-1: TIB-202, HL60: ACC 3, OCI-AML-3: DSMZ ACC-582, OCI-AML-5: DSMZ ACC-247) and cultured as per recommendations. MV-4–11 and HL-60 human AML cells were grown with RPMI-1640 (Gibco #11875135) supplemented with 10% heat inactivated serum. THP-1 human AML cells were grown with RPMI-1640 (Gibco #11875135) supplemented with 10% serum. OCI-AML-3 and OCI-AML-5 were cultured in 80% alpha-MEM (with ribo- and deoxyribonucleosides Gibco #12571063) with 20% heat inactivated serum. OCI-AML-5 were also supplemented with 10 ng/ml recombinant human GM-CSF (PeproTech #300–03). All the cell culture media also contained GlutaMAX (1:100, Gibco #35050061), Fetal Bovine Serum (cytiva SH30071.03I) and penicillin–streptomycin (1:100, Biological Industries #03–031-1B).

### Immunoblotting studies

Proteins were lysed with RIPA buffer containing proteases and phosphatase inhibitors from cell pellets. Western blot analysis was performed by means of standard techniques. Blots were incubated with antibodies detecting Cleaved Caspase 3 (1:750; Cell Signaling Cat#9661s), c-Myc (1:750; Cell Signaling Cat#9402), MCL-1 (1:1000; Cell Signaling Cat#5453), MDM2 (1:500; Cell Signaling #86934), PP2Ac (1:1000; Cell Signaling #2038), Phospho-Rpb1 CTD (Ser2; 1:1000; abcam #ab5095), Phospho-Rpb1 CTD (Ser5; 1:1000; Merck #04–1572), p53 (1:500; CM5; NCL-L-p53-CM5p) and phospho-Histone H2A.X (pSer139; 1:1000; Millipore #05–636). Secondary antibodies were Peroxidase-conjugated Goat anti-Rabbit (1:10,000; Jackson #111–035-144), Peroxidase-conjugated Rabbit anti-Goat (1:10,000; Jackson #705–035-003), and Peroxidase-conjugated Rabbit anti-Mouse (1:10,000; Jackson #315–035-003). Blots were developed using ECL (PerkinElmer 50–904-9323).

### Statistical Analysis

Dose-escalation was performed using a BOIN design. The overall population included all patients who received at least one dose of BTX-A51. Descriptive statistics were used for clinical, laboratory, pharmacokinetic and pharmacodynamic variables.

## Results

### Patients

Between January 13, 2020 and February 28, 2022, 31 patients were enrolled at the three participating centers. Among the patients, 28 had R/R AML and 3 patients had IPSS-R high-risk R/R MDS. Median age for the cohort was 75 years (22–84 years). Patients enrolled were heavily pretreated. The median number of prior lines of therapy was 2 (1–8) with 97% having received prior venetoclax and hypomethylating agents and 43% having no response after 2 lines of induction chemotherapy (primary induction failure). Additional baseline characteristics are summarized in Table 1.

### Treatment

**Figure 1:**
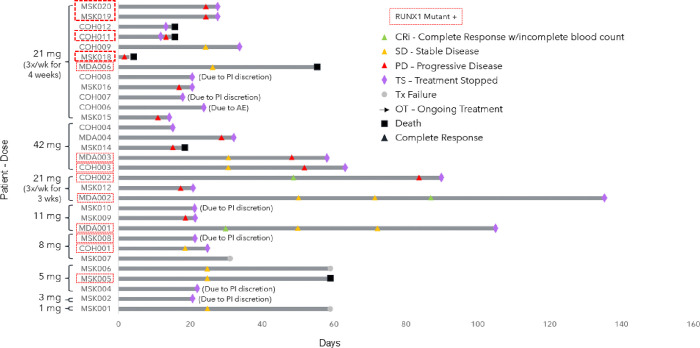
Swimmer plot of enrolled patients by dose level and their outcomes after treatment with BTX-A51, including time to response, duration of treatment, and patient status at time of data cutoff date. RUNX1-mutated patients are indicated with a red box. CRi, Complete response with incomplete count recovery, SD, stable disease, PD, progressive disease, TS, treatment stopped, Tx, treatment failure, OT, Ongoing treatment, mg, milligrams.

Dose levels administered ranged from 1 to 42mg. Treatment was given over 5 days per week for 21 days over a 28-day cycle (1mg dose), 3 days per week for 21 days over a 28-day cycle (3mg, 5mg, 8mg, 11mg, 21mg, 42mg doses), or 3 days per week for 28 days over a 28-day cycle (21mg dose). (Fig. 1.) Patients remained on treatment for a median of 24 days, range (3–135 days).

### Adverse Events and Dose Limiting Toxicities

A total of 31 (100%) patients receiving BTX-A51 had at least one treatment emergent AE of any grade (Supplemental Table 1). The most common treatment related adverse events (TRAE) occurring in ≥ 10% of participants were nausea (52%), vomiting (39%), hypokalemia (16%), diarrhea (13%), fatigue (13%), oral pain (13%), and increased alanine aminotransferase (ALT) (13%) (Table 2). TRAE grade 3 or higher were anemia (7%), hyperbilirubinemia (7%), acute hepatic failure 3%, increased AST (3%), increased ALT (3%), increased gamma-glutamyltransferase (3%), thrombocytopenia (3%), hypoxia (3%), and stomatitis (3%) (Table 3). Overall, 2 patients experienced an AE assessed by investigators as a DLT during cycle 1. One patient developed grade 3 alkaline phosphatase elevation at the 21 mg dose following treatment with BTX-A51. Another patient experienced grade 3 hepatic failure at the 42mg dose. All events promptly resolved after discontinuing study drug. The two patients experiencing a DLT were the only patients discontinuing BTX-A51 as a result of an adverse event. Although the criteria for the maximum tolerated dose was not met, the acute hepatic failure DLT led to discontinuation of the 42mg dose level. The recommended phase 2 dose was identified as 21mg administered 3 days per week for 28 days out of a 28-day cycle.

Overall, there were 11 deaths among patients during the study treatment and within 28 days of the last dose. Five of these deaths were attributed to disease progression. Six deaths occurred as a result of an adverse event determined to be unrelated to the study drug, including lung infection, cardiac arrest, intracranial hemorrhage, fungal infection, and septic shock.

### Pharmacokinetic and Pharmacodynamic Findings

**Figure 2:**
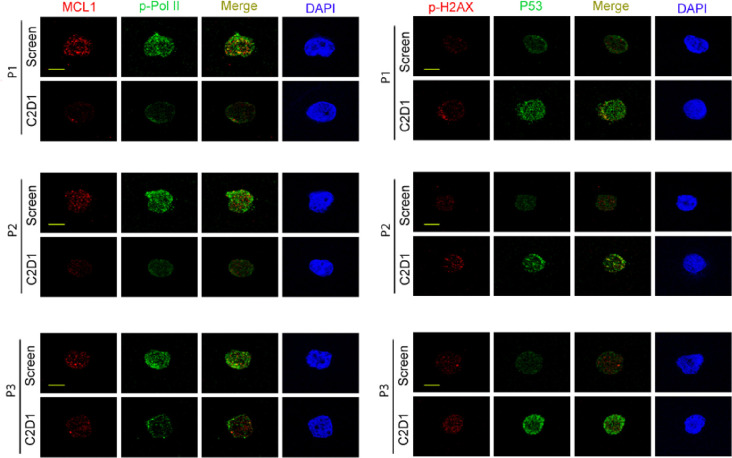
Pharmacodynamic studies of BTX-A51. Immunostaining of MCL1, P53, RNA polymerase II phosphorylation, and histone H2AX phosphorylation on CD34+ cells isolated from pretreatment (screening) and post-treatment (Cycle 2 Day1) bone marrow specimens. Reduced expression of MCL1 and RNA polymerase II phosphorylation and increased expression of P53 and histone H2AX phosphorylation after treatment with BTX-A51. Scale bar, 10μm.

BTX-A51 was rapidly absorbed with a mean time to maximum serum concentration (Cmax) between 4.5 and 6 hours across all dose levels (Supplemental Table 3). Plasma PK of BTX-A51 was dose-proportional between 1 and 42 mg with accumulation based on AUC between Day 1 and Day 5. The mean half-life was 28 hours (range, 17 to 45 hours, supplemental table 3). Interpatient variability was moderate, within a range typically observed for cancer patients, without obvious outliers. At the RP2D of 21mg 3 days /week for 28 days, the BTXA51 pre-dose concentrations were similar on cycle 1 day 15 and cycle 2 day 1 indicating steady-state levels. Serum macrophage inhibitory cytokine-1 levels, MIC-1, a secreted biomarker of p53 activation has been used as a pharmacodynamic marker of treatment in clinical trials of AML patients receiving MDM2 inhibitors.(30–32) MIC-1 levels were evaluable in 8 patients at the 21mg dose level. The serum MIC-1 fold change from baseline was variable on day 2 and remained elevated on day 5. (Supplemental Fig. 1). Immunocytochemistry studies were performed on CD34 + cells isolated from bone marrow aspirates collected from 3 patients treated at the 21mg and 42 mg dose levels, including a responder (Fig. 2). When compared to pre-treatment screening samples, post-treatment cycle 2 day 1 CD34 + bone marrow cells showed reduced expression of MCL1, polymerase II phosphorylation and increased expression of p53 and histone H2AX phosphorylation, a marker of DNA damage (Fig. 2).

### Clinical Efficacy

**Figure 3:**
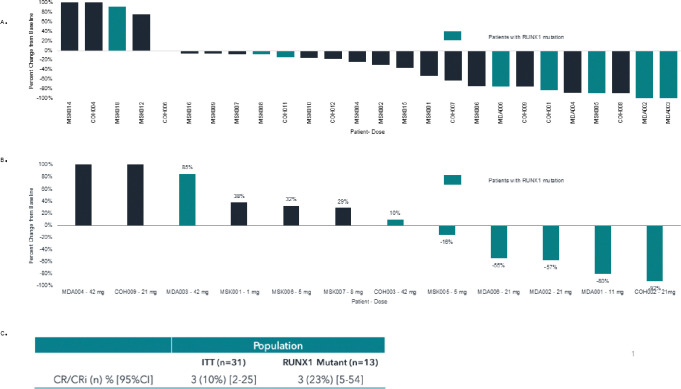
Bone marrow and peripheral blood blast count reduction after treatment with BTX-A51 by dose level and *RUNX1* mutation status. A. Peripheral blood blast count reduction during cycle 1 among patients with detectable peripheral blood blasts at screening. B. Best bone marrow blast reduction among patients with pre and post treatment bone marrow biopsies. C. CR/CRi rates among all patients in an intention-to-treat analysis and among all *RUNX1*-mutated patients. RUNX-1 mutated patients are identified by color in A. and B. CR, complete remission, CRi, complete response with incomplete count recovery, mg, milligrams, ITT, Intention to treat.

Overall, 3 out of 31 (10%) patients attained a complete remission with incomplete count recovery (CRi) in an intent to treat analysis. Among the three patients attaining CRi, BTX A51 was dosed at 11mg (n = 1) and 21mg (n = 2) (Figs. 1 and 3C). Two patients attained a response after the first cycle of treatment and 1 patient attained a CRi after the second cycle. The median duration of response was 1.9 months (range 1.5–2.5 months) and all three responding patients discontinued treatment after relapse (Fig. 1).

Among 26 patients with PB myeloblasts detected at baseline, PB blast count decreased at all dose levels in the majority of patients (85%) over the course of cycle 1 (Fig. 3A). Reductions from baseline in bone marrow myeloblasts as a best result occurred in 5 of 12 patients (42%) with available bone marrow biopsies, including 3 patients attaining a CRi and two patients with treatment failure (Fig. 3B).

Baseline mutational profiling was available in all 31 patients. *RUNX1* mutations were detected in 13 patients at the 5mg (n = 1), 8mg (n = 2), 11mg (n = 1), 21mg (n = 7) and 42mg (n = 2) dose levels (Fig. 1). CRi occurred in three out of 13 or 23% of *RUNX1*-mutated patients enrolled and three out of 10 or 30% of *RUNX1*-mutated patients receiving 11mg or higher of BTX-A51 (Fig. 3C). Seven out of eight *RUNX1*-mutant AML patients (88%) with PB blasts detectable at screening had a reduction in peripheral blasts during the first cycle of treatment with BTX-A51 (Fig. 3A). Additionally, all four patients with at least 50% reduction in bone marrow blast reduction had *RUNX1* mutations (Fig. 3B).

**Figure 4:**
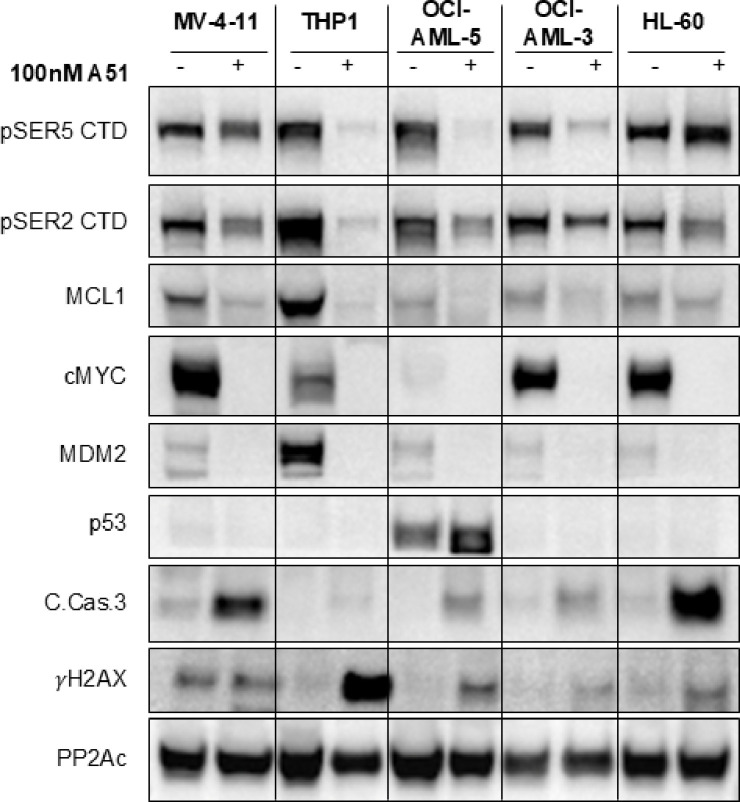
Immunoblot analysis of 5 different human AML cell lines treated with 100nM of A51 (+) or with DMSO (−) for 24 hours. PP2Ac is a loading control. Treatment with A51 resulted in decrease in phosphorylated Ser5 and Ser2 of the C-terminal domain (CTD) of RNA polymerase II, indicating inhibition of CDK7 and CDK9 respectively. This was followed by reduced expression of common leukemia oncogenes MYC, MDM2 and MCL1 and induction of a DNA damage response and apoptosis, as evidenced by the induction of γH2AX and cleaved caspase 3. At the indicated treatment procedure, a p53 response is evident only in OCI-AML-5 (RUNX1 mutant cell line).

**Figure 5:**
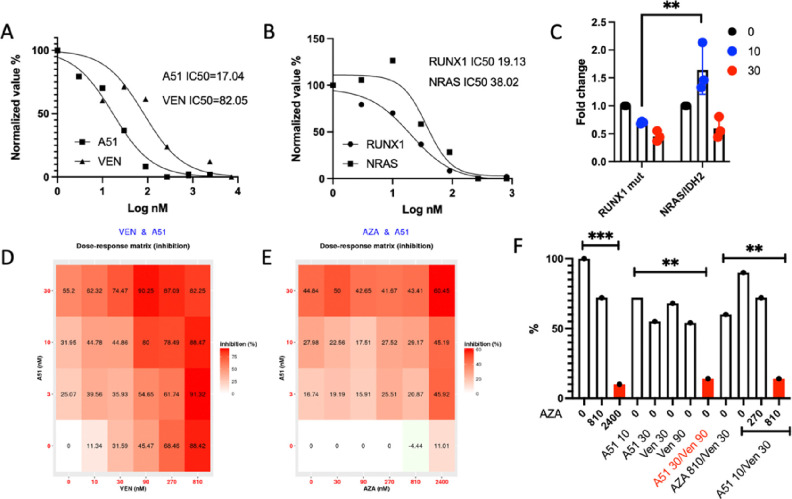
Ex vivo inhibition of primary *RUNX1*-mutated AML cells by BTX-A51. A. Flow sorted CD34 positive primary *RUNX1*-mutated AML blasts were plated onto preplated HUVEC_AD52 cells and the expansion of CD34 positive cells were calculated in the presence of BTX-A51 or Venetoclax. IC50 was calculated. B-C. *NRAS*/ *IDH2* mutant primary AML blasts were flow sorted and cultured in preplated HUVEC_AD52 cells. The expansion of CD34 positive cells were calculated in the presence of BTX-A51. IC50 was calculated. D-E. The additive effects of BTX-A51/Venetoclax and BTX-A51/Azacytidine were illustrated (Bliss synergy score was 8.04 and 8.75, respectively). F. The synergy between BTX-A51, Venetoclax and Azacytidine was plotted. *, p≤ 0.05; **, p ≤ 0.01; ***, p ≤ 0.001.

To further evaluate the anti-leukemic activity of BTX-A51 in *RUNX1 mutant* and *RUNX1 WT* AML, we performed in-vitro and ex-vivo studies with AML cell lines and primary patient samples. A51 treatment induced a significant reduction in Pol II CTD phosphorylation at Ser2 or Ser5 in *RUNX1* mutant (OCI AML-5) and *RUNX1* WT (MV4–11, THP-1, OCI AML-3, and HL-60) AML cell lines (Fig. 4). This resulted in a marked decrease in the expression of oncogenes such as MYC and MDM2. Importantly, this oncogene suppression, accompanied by the downregulation of the anti-apoptotic protein MCL1, led to the activation of p53 or DNA damage response and subsequent cleavage of caspase 3, thereby inducing leukemia cell apoptosis. A p53 response was only evident in the OCI-AML5 (*RUNX1* mutant cell line, Fig. 4). Flow sorted CD34 positive primary *RUNX1*-mutated AML blasts co-cultured with HUVEC_AD52 cells were more sensitive to BTX-A51 (IC50 17nM) than venetoclax (IC50 82nM) (Fig. 5A). Further, *RUNX1*-mutated CD34 + primary AML blasts cocultured with HUVEC-AD52 were more sensitive to BTX-A51 than *NRAS* and *IDH2 mutated* CD34 + primary AML blasts (IC50 19 nM vs. 38nM, p < 0.01, Fig. 5B–C).

As monotherapy, BTX-A51 showed promising but limited ex vivo effects on mutant *RUNX1* R/R AML cells. To study the additive/synergistic effects between BTX-A51 and venetoclax and/or azacitidine, we used the same HUVEC-AD52 coculture system to test the inhibitory effects on CD34 + leukemic blast expansion by single, double and triple combinations. BTX-A51 led to additive effects when combined with either venetoclax or azacitidine. (Fig. 5D–E). More importantly, in this ex vivo co-culture system, BTX-A51 30nM/Venetoclax 90nm showed maximal inhibitory effects. BTX-A51 30 nM/azacitidine 810nM had additive effects (Fig. 5E). However, in the presence of a low concentration of azacitidine (810 nM), the same maximal inhibitory activity was achieved with one-third the concentration of BTX-A51 and venetoclax. These results support BTX-A51/venetoclax combination or BTX-A51/venetoclax/azacitidine triple regimens in future clinical trial design.

## Discussion

Evasion of apoptosis, a hallmark of cancer, arises often as a result of inactivation of p53 and upregulation of pro-survival members of the BCL2 family (BCL-2, MCL-1, BCL-XL, BCL-W). Whereas p53 is mostly unmutated in AML, it is often inactive due to suppression by antagonistic factors, mostly Mdm2 and MdmX frequently amplified in AML.(16, 33) Attempts to inactivate Mdm2 inhibition as therapeutic means in AML are ongoing for many years with moderate success.(31, 32) Increasing p53 activity and suppressing expression of anti-apoptotic proteins represents a potential therapeutic strategy for improving outcomes in R/R myeloid malignancies and more broadly, other malignancies. In this first-in-human phase I study, treatment with BTX-A51, an oral CK1α and CDK7/9 inhibitor led to increased levels of MIC-1, a surrogate for p53 activation and suppression of *MCL1, MYC*, and *MDM2* expression. BTX-A51 was generally well-tolerated and demonstrated encouraging antileukemic activity, especially in AML harboring *RUNX1* mutations.

BTX-A51 was well-tolerated, in general, by the patient population at doses where responses were observed. The majority of TEAEs were grade 1 or 2, although two patients experienced treatment related DLTs with hepatic toxicity. Any grade treatment-related transaminase toxicity was observed in 7 patients and appeared dose-dependent, occurring more frequently at the highest 21mg and 42mg dose levels. At the RP2D, only 1 out of 12 patients had transaminase elevation. The most common treatment related adverse events were gastrointestinal toxicities (nausea, vomiting and diarrhea). Hypokalemia, fatigue, transaminase elevation, and hematologic toxicities were observed in a minority of treated patients. Gastrointestinal and hematologic toxicities have been observed in other studies with CDK9 inhibitors and CK1a degrading therapies (e.g. lenalidomide).(23, 25, 34) Notably, none of the patients receiving BTX-A51 experienced tumor lysis syndrome. The risk of TLS may have been mitigated by the eligibility criteria (WBC < 25K/uL and adequate renal function) and the intermittent dosing of BTX-A51.

In this study, the anti-leukemic activity of BTX-A51 was most apparent at higher doses. Although peripheral blast count reductions were observed at all dose levels, the three patients attaining a complete remission with incomplete count recovery (10%) received BTXA51 at 11mg or greater. Among patients at the recommended phase II dose of 21mg, we observed a CRi in 2 out of the 15 patients treated (13%). The response rates observed in this study are encouraging given the high-risk features of the cohort with 97% having received prior HMA and venetoclax and 41% having had prior induction failure.

Additionally, the anti-leukemic activity of BTX-A51 was enriched among patients with *RUNX1* mutations. *RUNX1* mutations occurred in all four patients with at least 50% bone marrow blast count reduction and all three responders. For the *RUNX1* cohort, the CR/CRi rate was 23% (3/13) and among those receiving at least 11mg or higher the CR/CRi rate was 30%. The increased sensitivity of *RUNX1* mutated leukemic cells to BTX A51 was also observed in primary AML cells harboring *RUNX1* mutations. *RUNX1* mutated primary patient AML cells were more sensitive to BTXA51 than venetoclax. Loss-of-function missense or nonsense mutations involving the *RUNX1* gene are common in MDS/ AML, occurring in 10–15% of patients and associated with inferior prognosis after treatment with hypomethylating agents and induction chemotherapy.(27, 35, 36) The poor outcomes are in part due to the lack of an approved therapy targeting *RUNX1*. The increased responses and anti-leukemic activity of BTX-A51 in *RUNX1*-mutated patients is encouraging and additional clinical trials evaluating BTX-A51 as a possible *RUNX1* targeting therapy as monotherapy and in combination are warranted.

In addition to *RUNX1*-mutated AML, BTX-A51 demonstrated activity in multiple other genetic mouse models of AML.(20) Although we did not observe responses among those without *RUNX1* mutations, BTX-A51 treatment decreased *MCL1* expression and increased levels of serum MIC-1, a biomarker of P53 activation. As inactivation of p53 and upregulation of other BCL-2 family of proteins represent mechanisms of resistance to BCL2 inhibition, these on-target effects of BTX-A51 provide rationale for combination treatment with venetoclax. Here, we demonstrated synergy when combining BTX-A51 with venetoclax and azacitidine providing further support for clinical studies evaluating these combinations.

In this study, BTX-A51 demonstrated tolerability with manageable side effects. BTX-A51 demonstrated on-target suppression of *MCL1* expression and activation of p53. Overall efficacy was modest but encouraging at higher doses, particularly among those patients with *RUNX1* mutated disease. Combinations with azacitidine and azacitidine and venetoclax are being explored.

## Figures and Tables

**Table 1:** Baseline characteristics of enrolled patients

Demographic/ Characteristic	Total Patients (N = 31)

Sex, n (%)	17 (55%)
Male	14 (45%)
Female	

Age, median (range)	75 (22–84)

Disease at study entry	28 (90%)
AML	3 (10%)
MDS, IPSS-R Higher risk	

Prior Lines of Systemic Therapy, median (range)	2 (1–8)

Prior Treatment with Venetoclax Regimen	31 (97%)

Prior Treatment with Hypomethylating Agent	31 (97%)

Primary Induction Failure	13 (41%)

Prior Allogeneic Hematopoietic Cell Transplants	3

*RUNX1*-mutated	12 (38%)

AML, Acute myeloid leukemia, MDS, Myelodysplastic syndrome, IPSS-R, revised international prognostic scoring system

**Table 2:** Treatment-Related Adverse Events with Incidence ≥ 10%

	Overall (N = 31)
**Preferred Term**	**n (%)**
Any Related TEAEs	26 (83.9%)
Nausea	16 (51.6%)
Vomiting	12 (38.7%)
Hypokalemia	5 (16.1%)
Diarrhea	4 (12.9%)
Fatigue	4 (12.9%)
Oral pain	4 (12.9%)
Alanine aminotransferase increased	4 (12.9%)

**Table 3:** Treatment-Related Adverse Events of grades 3 or 4

	Overall (N = 31)
**Preferred Term**	**n (%)**
Any Grade 3 or Higher Related TEAEs	6 (19.4%)
Anemia	2 (6.7%)
Blood bilirubin increased	2 (6.7%)
Aspartate aminotransferase increased	1 (3.2%)
Alanine aminotransferase increased	1 (3.2%)
Thrombocytopenia	1 (3.2%)
Acute hepatic failure	1 (3.2%)
Gamma-glutamyl transferase increased	1 (3.2%)
Hypoxia	1 (3.2%)
Platelet count decreased	1 (3.2%)
Stomatitis	1 (3.2%)
